# The Art of Mast Cell Adhesion

**DOI:** 10.3390/cells9122664

**Published:** 2020-12-11

**Authors:** Joanna Pastwińska, Paulina Żelechowska, Aurelia Walczak-Drzewiecka, Ewa Brzezińska-Błaszczyk, Jarosław Dastych

**Affiliations:** 1 Laboratory of Cellular Immunology, Institute of Medical Biology, Polish Academy of Sciences, 93-232 Lodz, Poland; joanna.pastwinska@op.pl (J.P.); adrzewiecka@cbm.pan.pl (A.W.-D.); 2 Department of Experimental Immunology, Medical University of Lodz, 92-213 Lodz, Poland; paulina.zelechowska@umed.lodz.pl (P.Ż.); ewab@csk.umed.lodz.pl (E.B.-B.)

**Keywords:** mast cell, adhesion, extracellular matrix, cell adhesion molecule, integrin, cadherin, selectin

## Abstract

Cell adhesion is one of the basic phenomena occurring in a living organism, affecting many other processes such as proliferation, differentiation, migration, or cell viability. Mast cells (MCs) are important elements involved in defending the host against various pathogens and regulating inflammatory processes. Due to numerous mediators, they are contributing to the modulation of many basic cellular processes in a variety of cells, including the expression and functioning of different adhesive molecules. They also express themselves many adhesive proteins, including ICAM-1, ICAM-3, VCAM-1, integrins, L-selectin, E-cadherin, and N-cadherin. These molecules enable MCs to interact with other cells and components of the extracellular matrix (ECM), creating structures such as adherens junctions and focal adhesion sites, and triggering a signaling cascade. A thorough understanding of these cellular mechanisms can create a better understanding of MC biology and reveal new goals for MC targeted therapy. This review will focus on the current knowledge of adhesion mechanisms with the involvement of MCs. It also provides insight into the influence of MCs or MC-derived mediators on the adhesion molecule expression in different cells.

## 1. Introduction

The process of eukaryotic cell adhesion has been studied for several decades. Already in the 1960s, studies on factors promoting cell adhesion referred to at that time as bridging agents, led to the development of the concept of the extracellular matrix (ECM) and specific adhesion receptors on cell surface capable of recognizing ECM [1]. Identification of the major families of adhesion receptors such as integrins and cadherins in the late 1970s to late 1980s paved the way to deciphering cell adhesion processes on a molecular level [2]. Now, it is generally accepted that the cell adhesion process is fundamental for the organization of tissues and cell migration and plays a critical role in a wide range of physiological phenomena, i.e., morphogenesis, wound healing [3,4], egg fertilization and implantation [1], and immune response [3].

Cell adhesion also plays an important role in the pathogenic processes. During tumorigenesis, the adhesive capacity of regular cells is highly reduced, which significantly affects the invasiveness of cancer cells and migration to other parts of the body, contributing to metastasis [3]. The main adhesion molecules that contribute to metastasis include selectins and integrins [5]. Interference with the adhesion process of eukaryotic cells is also one strategy of prokaryotic and viral pathogens invading multicellular organism cells. Bacteria have numerous adhesins that are able to recognize components of ECM such as fibronectin, collagen, laminin, or hyaluronic acid; and invasins, through which they are able to attach to integrin receptors of the host cells initiating a signal cascade that enables effective infection [6]. Similar mechanisms are utilized by viruses [7]. On the other hand, cell adhesion is essential for host immune response that depends on the network of the interaction of immune cells constantly migrating through the organism. The importance of cell adhesion for immune response is illustrated by congenital pathologic conditions resulting from abnormal cell adhesion known as leukocyte adhesion deficiency that is characterized by immunodeficiency leading to recurrent infections [8,9].

One type of immune cell are mast cells (MCs) that are derived from multipotent hematopoietic stem cells in the bone marrow, from which they migrate to the circulation and finally settle in various tissues as immature progenitors [10,11]. Maturation of MCs is occurring already in tissues where they closely interact with ECM and other cells. Stem cell factor (SCF) that is critical for the acquisition of mature MC phenotype is also upregulating integrin-mediated MC adhesion to the ECM component fibronectin [12,13]. At the same time, the adhesion of MCs to fibronectin is important for their interaction with fibroblasts that are expressing SCF and support the acquisition by MCs mature phenotype [14]. The critical role of integrin-mediated cell adhesion in homing of MC progenitors to their tissue location and their maturation, elegantly demonstrated in vivo in a mice knock-out model [15], has been reviewed by Collington et al. [16]. Furthermore, integrin-mediated MC adhesion to fibronectin upregulates MC activation and mediator release that is a cornerstone of MC proinflammatory activity [17,18]. Thus, adhesion of MC significantly influences different aspects of MC functions in the organism. The summary of the functional aspects of the expression of different adhesion receptors in MCs is presented in Table 1. In this article, we focus on MCs’ ability to express adhesion receptors, binding adhesion ligands, and forming intercellular junctions.

## 2. Mast Cells

Mature MCs are located predominantly surrounding blood vessels and nerves in most tissues and organs of the body. These cells are most abundant near surfaces exposed to the environment, i.e., in the respiratory, gastrointestinal, and genitourinary tracts as well as within papillary and reticular layers of the skin dermis [10,11].

The strong proinflammatory activity of MCs depends on MC activation and release of numerous potent mediators, which are divided into three categories. First of all, activation of MCs causes a release of mediators stored in cytoplasmic granules, such as histamine, proteoglycans, proteases, and certain cytokines and chemokines. This is followed by the generation of lipid mediators, including prostaglandins (PGs), leukotrienes (LTs), and thromboxanes (TXs), as well as the *de novo* production of cytokines, chemokines, and growth factors [42,43]. All MC-derived mediators significantly influence the activity and functions of other cells and tissues, which are in close proximity. Thus, apart from being markedly involved in IgE-mediated allergic reactions, MCs are also engaged in other pathogenic mechanisms such as cardiovascular disease, ischemia/reperfusion injuries, atherosclerosis [11], mastocytosis, asthma [44], or lymph node hypertrophy [45]. These cells are also crucial for the maintenance of body homeostasis through acting on wound healing, angiogenesis, and vascular permeability, and play an important role in innate and adaptive immunity [44,46], particularly in host defense against pathogens including bacteria, viruses, fungi, or some parasites [45]. MCs are widely recognized as important effector cells of inflammatory processes, as they affect distinct stages of inflammation, including its initiation and maintenance, but also its resolution. Hence, they participate in acute, chronic, as well as low-grade inflammation [47,48]. They are also implicated in the pathogenesis of several inflammatory disorders [49].

The phenotype of MC is defined by surface expression of many different receptors, with the high-affinity receptor for IgE (FcεRI) and c-Kit receptor for SCF being probably the most important. MCs also express several receptors for IgG, namely FcγRI, FcγRIIA, and FcγRIII, numerous receptors for arachidonic acid metabolites, including PGs, primarily PGE2 (i.e., EP_2_, EP_3_, EP_4_), LTs, mainly cysteinyl LTS [cysLTs (i.e., CYSLTR1, CYSLTR2, GPR17)], and LTB4 (i.e., BLT1R, BLT2R) [50,51,52], and receptors for histamine (i.e., H_1_, H_2_, H_4_). MCs receptors for cytokines include receptors for interleukins (i.e., IL-1R, IL-3R, IL-5R, IL-10R, IL-12R, IL-18R, growth factors [i.e., granulocyte-macrophage colony-stimulating factor (GM-CSF)R, transforming growth factor β (TGFβ)R] as well as for chemokines (i.e., CCR1, CCR3-5, CCR7, CXCR1-4, CXCR6, CX3CR1) [51,53]. On the MC surface, there are also receptors for complement components, i.e., C3a and C5a, neuropeptides [including substance P, nerve growth factor (NGF), and vasoactive intestinal peptide (VIP)] [11]. The last important group of MC receptors are pattern recognition receptors (PRRs) such as Toll-like receptors (TLRs), RIG-I-like receptors (RLRs), NOD-like receptors (NLRs), and C-type lectin receptors (CLRs) [54]. Interestingly, MCs also display certain inhibitory receptors, which upon MC activation may attenuate the generation of some proinflammatory mediators. This group comprises molecules such as CD172a, CD200R, CD300a, CD305, but also FcγRIIB, paired immunoglobulin-like receptor B (PIR-B), sialic acid-binding immunoglobulin-like lectins (Siglecs) as well as the platelet endothelial cell adhesion molecule (PECAM-1) [55].

## 3. Mast Cell Adhesion Receptors

### 3.1. Immunoglobulin Superfamily Cell Adhesion Molecules (IgSF CAMs)

Intercellular adhesion molecules (ICAMs), vascular cell adhesion molecule (VCAM-1), PECAM-1, and mucosal vascular addressin cell adhesion molecule 1 (MAdCAM-1) constitute a group of adhesion molecules that belong to the immunoglobulin superfamily [56,57,58,59]. MCs express ICAM-1, ICAM-3, and PECAM-1. Constitutive surface expression of ICAM-1 in the immature MC line HMC-1 was presented by Valent and co-workers as early as in 1991 [19]. Further studies identified the expression of ICAM-1 in mature human MCs isolated from the uterus, skin, and lungs [60], as well as in cultured MCs isolated from umbilical cord blood [20]. ICAM-3 expression has been shown in MCs isolated from human lungs and the HMC-1 line [22,60]. The PECAM-1 presence has been established on the surface of the murine peritoneal MCs (mPMCs) as well as in bone marrow-derived MCs (BMMCs) [61]. There are some data showing the inducibility of ICAMs expression in MCs. It has been reported that IL-4 [19,20], TNF, and IFN-γ [21] enhanced ICAM-1 expression in cultured human MCs isolated from cord blood and HMC-1 line. Numata et al. demonstrated that also IL-33 significantly increased ICAM-1 expression in BMMCs [62]. There are reports indicating that retinoic acid [63] and vitamin D [23] are capable of augmenting ICAM-3 expression in the HMC-1 line.

Adhesion molecules from the immunoglobulin family such as VCAM-1 and PECAM-1 expressed in endothelial cells play a crucial role in MC adhesion to endothelium mediated by very late antigen-4 (VLA-4/α4β1), α4β7, and αVβ3 integrins expressed on MC surface [24,25]. Moreover, Hallgren et al., established that VCAM-1 interactions with VLA-4 and α4β7 MC integrins are essential for murine MC progenitors recruitment [64]. It is also known that MC-derived mediators regulate the expression of ICAMs and VCAM-1 in various cells, including endothelial cells [65,66] and fibroblasts [67].

### 3.2. Integrins

Integrins are cell adhesion receptors that belong to the broadly understood CAMs group. Integrin receptors are heterodimers consisting of one α subunit and one β subunit that are non-covalently connected to each other. At present, 18 α subunits (α1-11, αIIb, αD, αE, αL, αM, αV, αX) and 8 β subunits (β1-8) are known and can create 24 functional surface receptors (α1β1, α2β1, α3β1, VLA-4, α5β1, α6β1, α7β1, α8β1, α9β1, α10β1, α11β1, αDβ2, lymphocyte function-associated antigen 1 (LFA-1/αLβ2), αMβ2, αXβ2, αIIbβ3, α6β4, α4β7, αEβ7, αVβ1, αVβ3, αVβ5, αVβ6, αVβ8). The α subunit determines the specificity for a particular ligand, while the β subunit is bound to the cytoskeleton of the cell and initiates a signaling cascade after receptor-ligand binding or conformational changes of the receptor [68,69].

Integrin ligand molecules are present in ECM and in cell surfaces, and in addition to integrin binding, they can recognize other ECM molecules, growth factors, cytokines, and proteolytic enzymes. Integrin receptors are often divided into four main groups depending on the recognized ligand. Thus, collagen receptors (α1β1, α2β1, α10β1, α11β1), laminin receptors (α3β1, α6β1, α7β1, α6β4), leukocyte-specific receptors (VLA-4, α9β1, αDβ2, LFA-1, αMβ2, α7β2, αXβ2, αXβ2, αXβ2), and receptors recognizing the RGD (tripeptide consisting of Arginine, Glycine, and Aspartate) sequence (α5β1, α8β1, αIIbβ3, αVβ1, αVβ3, αVβ5, αVβ6, αVβ8) used to be distinguished. However, an observation that a single integrin receptor recognizes multiple ligands and discovering additional integrin ligands made such classification impractical. Thus, integrin collagen receptors also include αXβ2, and integrin recognizing laminin are also α1β1, α2β1, and α10β1. Integrins recognizing fibronectin also include VLA-4, α4β7, and αDβ2. Vitronectin is recognized by α8β1, αDβ2, αIIbβ3, αVβ3, and αVβ5, while thrombospondin by α2β1, α3β1, VLA-4, α5β1, α6β1, αIIbβ3, and αVβ3. Integrin receptors also recognize ligands that were not taken into account in the original classification, such as E-cadherin (α2β1, αEβ7), heparin (αMβ2, αXβ2), angiostatin (α9β1, αVβ3), and others [68,69]. Besides anchoring the cell to the ECM or to other cells, binding of ligand by integrin receptor effects cytoskeleton and generate intracellular signals regulating proliferation, differentiation, survival or apoptosis, spreading, migration, and gene expression [69].

MCs express, to varying degrees, most of the existing integrin receptors. Significant expression of such integrin subunits as α4, α5, α6, αV, β1, and β5 was documented on the surface of the immature MC line [26]. To a lesser extent, α2 and α3 subunits were also expressed [26]. The expression of individual subunits may also change as MC mature. Thus, in addition to the α4, α5, β1 subunits, immature MCs express αL and β2 subunits. Upon maturation, MCs increase expression of α2, α3, α4, α5, αM, αV, αX, β1, β3, and decrease expression of αL and β2 subunits [70]. α4β7 integrin has been documented on MC progenitors, where it is playing a crucial role in cell homing to the gut [29]. Other integrins expressed by MCs include α7 [27], αIIb [33], αE [35], α9 [12,71], and β6 [38] subunits. We have not found sufficient literature data confirming expression of α1, α8, α10, α11, αD, β4, and β8 integrin subunits.

**Figure 1:**
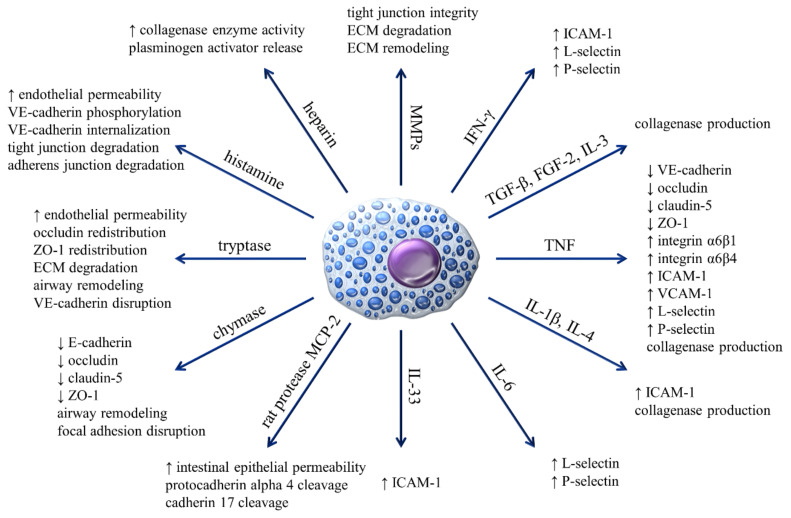
The influence of MCs and their mediators on the expression of adhesive molecules and regulation of processes related to the phenomenon of adhesion.

As already mentioned, the interaction of integrins with their ligands is involved in the homing of MC progenitors and, as presented in Table 1, regulates the function of mature MCs. Recognition and binding of fibronectin by VLA-4, α5β1, αVβ3 receptors enhances FcεRI-dependent activation of MCs, resulting in their greater degranulation. Mediators and cytokines released in higher amounts include β-hexosaminidase, TNF, IL-3, and IL-4 [30]. Similarly, the activation and degranulation of MCs were induced by the interaction of integrins containing the RGD sequence with fibrin, although this has no effect on MC viability [72]. Integrins LFA-1 binding ICAM-1 is mediating adhesion of BMMCs to activated T cells [73,74], and integrin receptors VLA-4 and LFA-1 are involved in the creation of immunologic synapsis between activated MCs and dendritic cells [75]. It is also known that MCs *via* numerous inflammatory mediators can regulate the expression of integrin receptors on other cell types (Figure 1). For example, MCs through TNF secretion are able to significantly increase the expression of α6β1 and α6β4 receptors on epidermal Langerhans cells that might have functional consequences on Langerhans cells interaction with locally recruited lymphocytes [76].

### 3.3. Selectins

Selectins comprise a group of type-I transmembrane glycoproteins that consist of an extracellular C-type lectin domain, an EGF-like module, and different numbers of consensus repeat (CR) domains. In the cell membrane, they are attached by a single transmembrane domain and a cytoplasmic tail. Selectins play an important role in regulating leukocyte recruitment during inflammation and have been involved in physiologic and pathologic processes such as angiogenesis [77], hematopoiesis [78], and tumor progression [79]. Among three known selectins, P-selectin is expressed in endothelial cells, megakaryocytes, thrombocytes, and macrophages [80,81,82]. The L-selectin expression is found in almost all leukocytes, and E-selectin is expressed solely in endothelial cells [83].

There is not much available data indicating selectin expression on MCs. Up to date, only Kaburagi et al. noted that L-selectin is highly expressed on the surface of mPMCs [39]. There are, however, evidence that selectins expressed in other cells are engaged in their adhesion to MCs by binding with selectin ligands on the MC surface. Steegmaier et al. demonstrated that endothelial P-selectin interaction with P-selectin glycoprotein ligand-1 (PSGL-1) on the MC surface plays an important role in BMMC adhesion to the endothelium [84]. This observation was confirmed by studies conducted by Dudeck et al., in which rolling and diapedesis of BMMCs have been observed [25]. These observations suggest that binding of endothelial selectins to MCs selectin ligands might be important for the process of accumulation of MCs in inflammatory sites, in which they are passing the endothelial barrier. As presented in Figure 1, there are many reports on regulation by MCs expression of selectins in other cells such as endothelial cells [65,85,86,87], B cells [88], and neutrophils and monocytes/macrophages [89].

### 3.4. Cadherins

Cadherins constitute a superfamily of adhesion molecules that are most commonly found on epithelial, endothelial, or nerve cells. Due to their number, structural differences, and performed functions, they were divided into four groups: (I) Classical cadherins, (II) desmosomal cadherins, (III) protocadherins, and (IV) unconventional/ungrouped cadherins. Classical cadherins include E-cadherin (*CDH1*), N-cadherin (*CDH2*), and P-cadherin (*CDH3*). Within desmosomal cadherins, desmogleins (*DSG1*, *DSG2*, *DSG3*, *DSG4*), and desmocollins (*DSC1*, *DSC2*, *DSC3*) are most commonly expressed by epithelial cells [90]. The most numerous groups are protocadherins, with more than 60 members mostly expressed in the central nervous system [91]. Cadherins play an important role in adhesion through the reduction of interfacial tension and stabilization of cell interactions during adherens junctions. At the same time, they can also influence morphogenesis, cell polarity, and proliferation [92].

Scarce data indicate the presence of cadherin in the MC membrane, and those that exist concern E-cadherin and N-cadherin. Nishida et al. reported expression of E-cadherin and β-catenin in human immature MC line, suggesting that MCs may be able to form adherens junctions similarly to epithelial cells [40]. Such a hypothesis was confirmed by the expression of E-cadherin and α- and β-catenins as well as the formation of the functional complex observed in murine MCs [41]. N-cadherin expression in MCs has also been documented [41], and MC N-cadherins were contributing to the creation of synapse-like structures with nerve cells [93]. Contrary to reports of E-cadherin and N-cadherin expression in MCs, the study of P-cadherin expression showed the absence of P-cadherin mRNA and protein in BMMCs and mPMCs [41]. 

MCs mediators such as tryptase and histamine have the ability to downregulate cadherin expression, structure, and functioning, increasing endothelial permeability [94,95,96] (Figure 1).

## 4. Mast Cells and Extracellular Matrix

ECM is filling the space between cells serving as a scaffold for the creation of supracellular structures and cell migration. The importance of ECM can be clearly seen on the basis of the number of various pathological conditions that result from irregularities in the structure or function of ECM components [97,98]. The main components of ECM are water, proteins, and polysaccharides. Adhesion receptors that interact with ECM components are integrins, discoidin domain receptors (DDRs), syndecans, and CD44 [97].

One of the most important components of ECM are proteoglycans (PGs) such as hyalectans, aggrecan, versican, neurocan, brevican, and decorin. They may interact with various growth factors, proinflammatory mediators, cell receptors, and other ECM components by regulating basic cellular processes, i.e., proliferation, differentiation, apoptosis, migration, and adhesion. They also contribute to the continuous remodeling of the ECM. Another important element of ECM is hyaluronic acid glycosaminoglycan, characterized by high plasticity due to its ability to bind water molecules. ECM proteins include collagens, which are the most numerous among all ECM proteins, elastin, fibronectin, laminin, and vitronectin [99,100]. Moreover, components of ECM include numerous proteolytic enzymes, which comprise matrix metalloproteinases, plasminogen/plasmin, and cathepsin proteases [99].

There are many literature reports documenting MC adhesion to various ECM components, including fibronectin, vitronectin, laminin, and hyaluronic acid. Interestingly, there are no reports unequivocally demonstrating MC adhesion to the major ECM protein that is collagen. Adhesion of MCs to ECM proteins is mediated by integrin receptors, while in the case of hyaluronic acid, the CD44 receptor is involved in ligand binding [37,101,102,103]. As already stated, MC adhesion to ECM regulates MC functions such as the release of MC mediators. In addition to the fact that MCs can interact directly with the ECM, they can also affect it through numerous mediators and proteolytic enzymes that are stored in MC granules and released after degranulation, contributing either directly or indirectly to ECM degradation [104,105,106] and remodeling [107,108] (Figure 1).

## 5. Mast Cells and Intercellular Junctions

**Figure 2:**
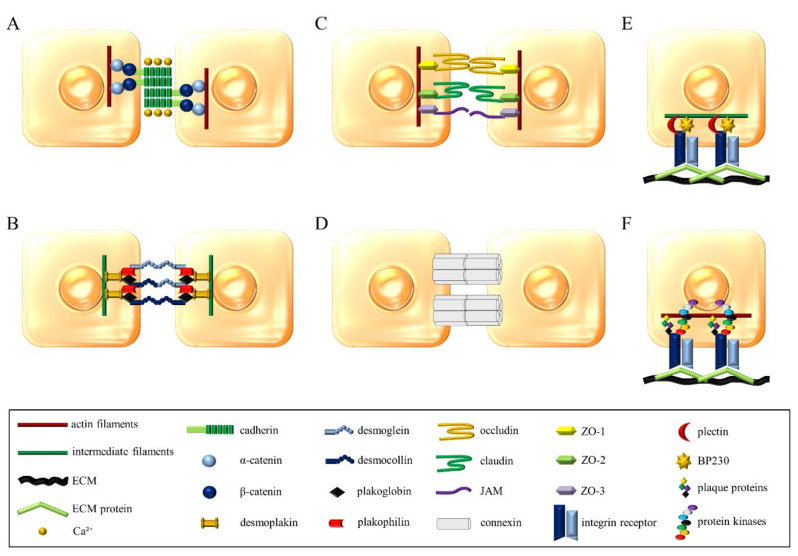
Principal interactions of cell-cell junctions: (**A**) Adherens junctions, (**B**) desmosomes, (**C**) tight junctions, (**D**) gap junctions; and cell-ECM junctions: (**E**) Hemidesmosomes, (**F**) focal adhesions.

Cadherins are critical for the formation of adherens junctions that are probably the most important intercellular connections necessary to maintain solid tissues. The structure created by cadherins in adherens junctions has been referred to as "zipper" (Figure 2A) [4,109,110]. As already mentioned, MCs express cadherins, but the functional significance of this expression is not clear. On the other hand, MCs, through their numerous mediators, downregulate stability and formation of adherens junctions in other cells [111,112,113] (Figure 1). Another type of junctions are desmosomes, most abundant in epithelial and cardiac muscle cells. Likewise, adherens junctions, desmosome-forming proteins belong to the cadherin family, although they are referred to as desmogleins and desmocollins (Figure 2B) [4,110,114]. The only report connecting MCs with desmosomes is an electron microscopy-based study of MCs in swine kidney that revealed the presence of desmosome-like junctions between MCs and epithelial cells [115].

Another type of cell junction are tight junctions, also known as occluding junctions or zonula occludens that create an impermeable barrier and determines whether the site between connecting cells will be selectively permeable to ions and macromolecules [4]. Claudins and occludin primarily participate in these cell junctions as the transmembrane components (Figure 2C) [110,116]. There are no reports on the expression of claudins and occludins and the formation of tight junctions by MCs. As in the case of adherens junctions, MC mediators downregulate tight junctions formed by other types of cells [111,112,113,117] (Figure 1).

Gap junctions are arranged from a transmembrane protein called connexin, which combines to form hexamers in the shape of a tunnel. This structure is called a connexon. When present on one cell, it attaches to a similar structure on a neighboring cell, enabling the direct and selective flow of ions and small molecules (Figure 2D) [118]. Mouse MCs express connexins, specifically Cx43 and Cx32, at both mRNA and protein levels, while Cx43 was also detected on the MC surface [119]. There are also reports regarding MCs communicating with other cells *via* gap junctions. Such connections between MCs and fibroblasts were first observed in 1995 in the isolated avian eye [120]. Formation of gap junction between MCs and fibroblasts was confirmed in vitro using BMMCs cocultured with murine fibroblast line [119] and rat MC line with human primary fibroblast [121]. There are suggestions that the formation of the gap junction between MCs and fibroblasts facilitates the effects of fibroblasts on the MC phenotype. On the other hand, gap junctions can be engaged in effects of MCs proliferation and phenotype of fibroblasts observed in vitro [120,121]. There is also a report regarding the formation of gap junctions between mouse mastocytoma and endothelial cell lines associated with increased angiogenesis activity of endothelial cells [122].

Other types of adhesive connections that may arise are those between cells and the ECM recognized by cellular integrins. An example of such a connection are hemidesmosomes, in which integrin α6β4 recognizes laminin in the basement membrane (Figure 2E) [4,123]. Since there are no reports in the literature on the expression of β4 integrin subunit in MCs and our own research confirmed this [12], thus formation of hemidesmosomes by MC seems to be unlikely.

Integrins also mediate the formation of other junctions between cells and ECM known as focal adhesion sites (Figure 2F). The formation of focal adhesion sites plays an important role in cell motility [4,124]. Literature data indicate that the formation of focal adhesions by MC may be associated with the secretion of inflammatory mediators because, after stimulation with various activating agents, redistribution of vinculin and talin into MCs cytoskeleton fraction can be observed [125]. On the other hand, MC degranulation, specifically the secretion of chymase, affects the disruption of focal adhesions arising between the ECM and other cells such as smooth muscle cells [126] (Figure 1).

## 6. Adhesion Related Signaling Pathways

Regulation of adhesive processes is of prime importance for basic physiological processes that are constantly taking place in the human body. Molecules involved in adhesion signaling pathways are most often associated with other pathways forming a network of interrelationships. Consequently, regulation of adhesion significantly affects cell proliferation, differentiation, and survival. Moreover, adhesion is inseparably connected with cell motility, thus, contributing to the migration of immune cells towards an ongoing inflammatory response or pathogen, but on the other hand, it can also positively regulate the rate of oncogenesis. Integrin-related signaling pathways are one of the best-studied signaling processes related to cell adhesion. It is known that intracellular signals regulate integrin-mediated adhesion by changing integrin conformation that leads to changes in its binding affinity [4]. This process, called inside-out integrin signaling, enables integrins to recognize and bind to specific ligands. Molecular mechanisms of inside-out integrin signaling involve cytoskeletal proteins containing in their sequence the FERM domain, such as talin and kindlin. FERM domain interacts with the first NPxY motif (membrane proximal) of integrin β subunit, disrupting the salt bridge between α and β subunits, causing tail separation and change in integrin receptor conformation [127,128,129]. Initiation of inside-out integrin signaling depends on specific mediators, including proinflammatory cytokines, and chemokines may activate integrins by binding to their specific receptors and initiation of intracellular signals. 

The binding of integrin receptor to its ligand initiates in turn a different signaling process called integrin outside-in signaling that regulates various vital cellular processes, including those related to immune functions. The outside-in integrin pathway involves a number of signaling proteins including tyrosine kinases from Src and Syk kinases family, focal adhesion kinase (FAK), Ras and Rho GTPase, and adapters such as Cas/Crk, and paxillin that interact creating macromolecular structures, such as focal adhesions or podosomes [127]. The engagement of the integrin receptor with its ligand triggers phosphorylation of Src and Syk kinases that phosphorylate downstream molecules, including FAK. Activated FAK stabilizes the conformational structure of Src that allows for maintaining its catalytic activity and leads to activation of the Ras-MEK-MAPK pathway [128,130,131].

Similar to integrins, other adhesion receptors are also regulated by intracellular signals and initiate such signals upon binding to their ligand. For example, hyaluronan receptor CD44 affinity is regulated by certain intracellular signaling pathways, and binding of hyaluronic acid by CD44 induces phosphorylation of c-Src, Rac1, and RhoA [132]. There is also an example of cross-talk between signaling pathways related to different types of adhesion molecules. Thus, during the process of leucocyte rolling on endothelium selectin binding, their ligand initiate signaling pathways downregulating integrin affinity to their ligands [133].

Many of the above signaling pathways, as well as others, play an important role in regulating MC adhesiveness and in adhesion mediated effects on MC functions. Adhesion of MCs to fibronectin is enhanced upon activation by antigen/IgE *via* FcεRI receptor as a result of signaling cascade involving SFKs, Syk, Lyn, Hck, Btk, and protein kinase C (PKC) [134,135]. SCF *via* its receptor, c-kit, upregulates MC adhesion to fibronectin. This growth factor is also critical for the maturation and survival of MCs [13,135,136]. Genistein, which is a highly specific inhibitor of protein tyrosine kinase (PTK), only partially inhibits SCF-mediated adhesion to fibronectin while PI3K inhibitor wortmannin inhibits it completely, which suggests that this process depends not only on PTK activity of c-kit but also engages PI3K [137]. Other activators of MC adhesion to fibronectin include aggregated IgG [138], serum amyloid A [139] Fps/Fes PTK [136], thrombin, and protease-activated receptor-1 (PAR-1). MC adhesion to fibronectin is sensitive to pertussis toxin, wortmannin, calphostin, U0126, SB203580, which are inhibitors of Gi proteins, PI3K, PKC, MEK 1/2, AND p38 MAPK, respectively, indicating the involvement of these molecules in a signaling pathway regulating MC adhesion [140]. There is also evidence of regulation of MC adhesion to fibronectin by the SWAP-70 pathway [141].

## 7. Conclusions

MCs adhesion plays an important role in different aspects of MCs biology, including homing of MCs progenitors to tissues, MCs maturation, and activation by specific signals. While MCs express adhesion receptors that belong to different families of proteins (Table 2), the greatest impact on their immune functions could be similar to other immune cells attributed to integrins (Table 1). MCs express multiple integrins, and the activity of these receptors is upregulated by different MC activators, including antigen/IgE and SCF. Upregulation of MCs integrin function results in upregulation of MCs adhesion to ECM, and to other cells, including immune cells, such as T cells and dendritic cells. Binding of MC integrins to their ligands in ECM or on the surface of other cells, in turn, upregulates release from MCs potent proinflammatory mediators. Thus integrin-mediated MC adhesion seems to be essential for enabling MC to perform their functions in defense of host against pathogens and regulation of inflammatory processes. The functional role of other than integrin MC adhesion receptors such as cadherins and connexins is less understood. However, the evidence of formation by MCs with other cells such as fibroblasts and endothelial cells gap junctions correlated with phenotypic changes in both interacting cell types suggests the importance of connexins for MC function in vivo. It is worth noticing that MCs are not only able to adhere to other cells and form with them intercellular junctions, but they are also capable of regulating the expression of CAMs and their ability to form intercellular junctions in other cells (Figure 1). Although much information supporting the important role of MC adhesion for their physiological and pathological roles in the organism is already available, there are still significant gaps in our knowledge on this subject. A better understanding of molecular mechanisms of MC adhesion can create new opportunities and reveal new goals for MC targeted therapy.

## Figures and Tables

**Table 1:** Cell adhesion receptor functions in mast cells (MCs).

CAMs	Functions in MCs		References
	Ligand/-s	Action	
IgSF CAMs			
ICAM-1	LFA-1 (αLβ2)	Migration of MCs to inflamed tissues. Interaction with stroma cells, cytotoxic or helper T cells, Langerhans’ cells, monocytes, and granulocytes. Activation of lymphocytes.	[19,20,21]
ICAM-3	αDβ2 LFA-1 (αLβ2)	Regulation of proliferation, apoptosis, spreading, and cytokine production. Cross-linking leads to homotypic/heterotypic aggregation and MC adhesion to ECM.	[22,23]
VCAM-1	VLA-4 (α4β1) α4β7	Scarce data.	[24]
PECAM-1	αVβ3	Cross-talk with PECAM-1 expressed on endothelial cells leads to transmigration of BMMCs through skin endothelial cell barrier.	[25]
Integrins			
α2β1	collagen laminin	Scarce data.	[26]
α3β1	laminin fibronectin	Interaction with ECM. Contribution to human MC migration.	[27,28]
VLA-4 (α4β1)	VCAM-1 MAdCAM-1 fibronectin	Recruitment of MC progenitors in allergic inflammation. Recruitment of mouse MC progenitors to inflamed lungs. Interaction with ECM proteins. Mouse and rat MC activation and degranulation associated with the high-affinity IgE receptor cross-linking.	[24,28,29,30]
α4β7	VCAM-1 MAdCAM-1	Recruitment of MC progenitors in allergic inflammation. Homing of MC progenitors to small intestine. Recruitment of mouse MC progenitors to inflamed lungs.	[24,25,29,31]
α5β1	fibronectin	Interaction with ECM proteins. Mouse and rat MC activation and degranulation associated with the high-affinity IgE receptor cross-linking.	[28,30]
α6β1	laminin	Presence on BMMC and their mature form – CTMC, thus, may be related to MC heterogeneity and involved in MC development.	[32]
α7β1	laminin	Interaction with ECM proteins. Present mainly in mouse.	[27]
α9β1		Scarce data.	
αIIbβ3	fibronectin vitronectin, fibrinogen	Possible role in differentiation and homing of human and mouse tissue MCs. Activation of MCs.	[33,34]
αEβ7	E-cadherin	Heterophilic adhesion of BMMCs to epithelial cells. Possible contribution to allergic inflammation and removal of nematodes.	[31,35]
LFA-1 (αLβ2)		Function not clear. Possible role in MC-other inflammatory cell interaction and immature MC homing.	[24,36]
αMβ2		Function not clear. Possible role in MC-other inflammatory cell interaction and immature MC homing.	[24,36]
αVβ1	vitronectin	Clustering of the receptor on filopodia during cell spreading.	[37]
αVβ3	PECAM-1	Rolling of immature MCs during inflammation. Transmigration of BMMCs through skin endothelial cell barrier. Mouse and rat MC activation and degranulation associated with the high-affinity IgE receptor cross-linking.	[25,30]
αVβ5	vitronectin	Clustering of the receptor on filopodia during cell spreading.	[37]
αVβ6		Participation in MC protease expression regulation, thus, may regulate airway responsiveness in allergic asthma.	[38]
αXβ2		Function not clear in the context of MC adhesion. Possible role in MC-other inflammatory cell interaction and immature MC homing.	[24,36]
Selectins			
L-selectin		Recruitment of mouse MCs during cutaneous Arthus reaction.	[39]
Cadherins			
E-cadherin	E-cadherin	Homophilic adhesion of BMMCs and HMC-1 to E-cadherin on epithelial cells. Possible contribution to allergic inflammation and removal of nematodes. Possible role in differentiation, proliferation and cell recognition.	[35,40]
N-cadherin		Present in BMMC. Exact role not clear.	[41]

BMMCs, (murine) bone marrow-derived MCs; CTMC, (murine) connective tissue MCs; HMC-1, human MC line; ICAM, intercellular adhesion molecule; IgSF CAMs, immunoglobulin superfamily cell adhesion molecules; LFA-1, lymphocyte function-associated antigen 1; MAdCAM-1, mucosal vascular addressin cell adhesion molecule 1; PECAM-1, platelet endothelial cell adhesion molecule; VCAM-1, vascular cell adhesion molecule; VLA-4, very late antigen-4.

**Table 2:** Cell adhesion molecule expression in various types of MCs.

CAMs	Expression in MCs			References
	Primary and In Vitro Differentiated Cells		Cell Lines	
	Mature	Immature	(Immature)	
IgSF CAMs				
ICAM-1	LMCs (h), SMCs (h), rPMCs (r)	CBMCs (h), BMMCs (m)	HMC-1 (h), MC-9 (m)	[20,62,142,143]
ICAM-3	LMCs (h), SMCs (h)		HMC-1 (h)	[22]
VCAM-1		BMMCs (m)		[144]
PECAM-1	mPMCs (m)	BMMCs (m)	RBL-2H3 (r)	[61,145]
Integrins				
α2	mPMCs (m)	CBMCs (h), BMMCs (m)	HMC-1 (h), RBL-2H3 (r)	[26,34,71,102,146]
α3	SMCs (h)	CBMCs (h), BMMCs (m)	HMC-1 (h), LAD2 (h)	[12,26,28,71,102]
α4	LMCs (h), SMCs (h), mPMCs (m), rPMCs (r)	CBMCs (h), BMMCs (m)	HMC-1 (h), LAD2 (h), RBL-2H3 (r)	[12,18,26,28,30,34,70,147]
α5	LMCs (h), SMCs (h), mPMCs (m), rPMCs (r)	CBMCs (h), BMMCs (m)	HMC-1 (h), LAD2 (h), MC-9 (m), RBL-2H3 (r)	[12,18,26,27,28,30,70,147]
α6	SMCs (h), mPMCs (m), rPMCs (r)	BMMCs (m)	HMC-1 (h), MC-9 (m), RBL-2H3 (r)	[18,26,27,32,146,148]
α7		BMMCs (m)	LAD2 (h)	[12,27]
α9	mPMCs (m)	CBMCs (h), BMMCs (m)	HMC-1 (h), LAD2 (h)	[12,71,147,149]
αIIb	mPMCs (m)	CBMCs (h), BMMCs (m)		[33,34]
αE	mPMCs (m)	BMMCs (m)		[35,150]
αL	SMCs (h)	CBMCs (m), BMMCs (m)	HMC-1 (h), LAD2 (h), MC-9 (m)	[12,34,36,70,142,151]
αM	LMCs (h), mPMCs (m)	CBMCs (h), BMMCs (m)	HMC-1 (h), LAD2 (h)	[12,36,70,147,149,152]
αV	LMCs (h), SMCs (h), rPMCs (r)	CBMCs (h), BMMCs (m)	HMC-1 (h), LAD2 (h), RBL-2H3 (r)	[18,26,30,38,71,153]
αX	LMCs (h), SMCs (h)	CBMCs (h)	HMC-1 (h), LAD2 (h)	[12,36,70,152]
β1	LMCs (h), SMCs (h), mPMCs (m), rPMCs (r)	CBMCs (h), BMMCs (m)	HMC-1 (h), LAD2 (h), MC-9 (m), RBL-2H3 (r)	[12,18,26,34,71,146,148]
β2	LMCs (h), SMCs (h), mPMCs (m), rPMCs (r)	CBMCs (h), BMMCs (m)	HMC-1 (h), RBL-2H3 (r)	[34,36,39,70,143,151,152,154]
β3	LMCs (h), SMCs (h), mPMCs (m), rPMCs (r)	CBMCs (h), BMMCs (m)	HMC-1 (h), LAD2 (h), RBL-2H3 (r)	[18,30,34,71,153]
β5			HMC-1 (h), LAD2 (h)	[26,153]
β6		BMMCs (m)		[38]
β7	mPMCs (m), rPMCs (r)	BMMCs (m)	LAD2 (h), RBL-2H3 (r)	[12,35,146,155]
Selectins				
L-selectin	mPMCs (m)	BMMCs (m)		[39,156]
Cadherins				
E-cadherin	mPMCs (m)	BMMCs (m)	HMC-1 (h)	[35,40,41]
N-cadherin		BMMCs (m)		[41]

BMMCs, (murine) bone marrow-derived MCs; CBMCs, (human) cord blood-derived MCs; HMC-1, human MC line; ICAM, intercellular adhesion molecule; IgSF CAMs, immunoglobulin superfamily cell adhesion molecules; LAD2, Laboratory of Allergic Diseases 2 (human MC line); LMCs, (human) lung MCs; MC-9, murine MC line; mPMCs, murine peritoneal MCs; PECAM-1, platelet endothelial cell adhesion molecule; RBL-2H3, rat basophilic leukemia clone 2H3; rPMCs, rat peritoneal MCs; SMCs, (human) skin MCs; VCAM-1, vascular cell adhesion molecule. (h), human; (m), mouse; (r), rat; underlined types of MCs represent only gene expression.
